# 4077 Listening with the HEAR-QL: Quality of Life in Children with Hearing Loss

**DOI:** 10.1017/cts.2020.410

**Published:** 2020-07-29

**Authors:** Brandon Malik Wahba, Judith Lieu

**Affiliations:** 1 Washington University in St. Louis, Institute Of Clinical and Translational Sciences; 2 Washington University School of Medicine

## Abstract

OBJECTIVES/GOALS: This study evaluates the utility of self-reported quality of life measure in children with hearing loss. We will compare self-reported HEAR-QL scores with parent-reported HEAR-QL scores. We will then test the relationship between HEAR-QL scores and scores on a standardized assessment of cognition, the NIH Cognition Battery. METHODS/STUDY POPULATION: We will administer the HEAR-QL questionnaire to children with hearing loss and their parents. We will then administer the NIH Cognition Battery to the child. We will include in our population children ages 7 to 14 with hearing loss of any severity or side. We will exclude those with intellectual disability, disorders of speech or language, or those who would be unable to complete the questionnaires for any reason. Children will be recruited from Otolaryngology clinics at St. Louis Children’s Hospital based on ICD diagnosis of sensorineural hearing loss between 01/2015 – 03/2020. RESULTS/ANTICIPATED RESULTS: We will aim to recruit 44 patients in total, which is the sample size needed to detect a moderate correlation (r = 0.4) with a 1-sided α = 0.05 and 1-β = 0.8. HEAR-QL scores and NIH Cognition Battery scores will be reported using descriptive statistics. Linear regression as well as correlation analysis between HEAR-QL scores and cognitive testing scores will be performed using a 1-sided α = 0.05, with 1-β = 0.8. If recruitment is sufficient, we will adjust for demographics that are significantly correlated with the outcome on multivariate analysis. Finally, we will test for agreement between parent report and child report by calculating a Kappa statistic. DISCUSSION/SIGNIFICANCE OF IMPACT: There is little clarity on the necessity of amplification in children with hearing loss, yet the child’s perspective is not routinely assessed in clinical practice. This study employs self-report in a pediatric population with hearing loss to find out if children provide new and reliable information.

